# Critical Incidents for Hispanic Students on the Path to the STEM Doctorate

**DOI:** 10.3389/fpsyg.2022.734307

**Published:** 2022-03-15

**Authors:** Dawn Horton, Irma Torres-Catanach

**Affiliations:** ^1^Department of Student Development, University of Massachusetts, Amherst, MA, United States; ^2^College of Engineering, University of Texas at El Paso, El Paso, TX, United States

**Keywords:** Hispanic, STEM, doctorate, graduate, career decision making, Latinx

## Abstract

Hispanics are grossly underrepresented in the receipt of STEM Ph.Ds. The National Science Foundation (NSF) Science and Engineering Indicators (Trapani and Hale, 2019) suggest that only 7.8% of S and E doctoral recipients are Hispanic while their representation in the population is more than twice that, and that figure goes even higher if restricted to those within the college-age range. To address this gap, the NSF has awarded a grant (the Hispanic Alliance for Graduate Education and the Professoriate, H-AGEP) to the City College of New York and the University of Texas at El Paso to work with Hispanic STEM doctoral students to provide teaching training and preparation for academic positions so they can become role models for Hispanic community college undergraduates. In working to understand the career-decision making of our Fellows, in-depth interviews were conducted (*n* = 13) to understand what put them on the path to defy the odds and become a STEM doctoral recipient. Interview results suggest that isolated, critical incidents and chance events were responsible for a number of our students entering into doctoral programs. This research suggests that for some Hispanic STEM doctoral students the experience of chance events meant the path to a STEM doctorate was not assured from a young age and further, that the provision of “planned” critical incidents may support an increase in Hispanic STEM doctoral enrollment.

## Introduction

Hispanics are grossly underrepresented in the receipt of STEM Ph.Ds. The National Science Foundation (NSF) Science and Engineering Indicators (Trapani and Hale, 2019) suggest that only 7.8% of S and E doctoral recipients are Hispanic while their representation in the population is more than twice that, and that figure goes even higher if restricted to those within the college-age range. The underrepresentation of Hispanics with Ph.Ds. in STEM fields raises questions as to why this occurs. The career path to a Ph.D. in STEM can be considered to have three parts: the development of an interest in STEM, education in STEM, and the decision to engage in a career in STEM. The objective of this research is to illuminate the first two parts of this pathway among Hispanic STEM doctoral students. As part of an NSF funded grant (the Hispanic Alliance for Graduate Education and the Professoriate, H-AGEP) the City College of New York and the University of Texas at El Paso have worked with Hispanic STEM doctoral students to provide preparation for possible academic careers, particularly at community colleges. In-depth interviews were conducted with all participants in the first two cohorts (*n* = 13) to document how they developed their interest in STEM and came to study at the Ph.D. level.

Interview results suggested that isolated, critical incidents and chance events were responsible for a number of our students becoming engaged with STEM and ultimately entering into doctoral programs. These seemingly chance events suggest that the path for many Hispanic STEM doctoral students is not one that is assured from a young age. Hispanic student participation in STEM activities and attainment of STEM degrees may increase with more “planned” critical incidents that would support their pursuit of STEM career pathways.

## Literature Review

### Hispanic Representation in STEM

The United States Census Bureau reported that in August of 2012, Hispanics had become the “nation’s largest ethnic or racial minority” during the 2011 calendar year (U.S. Census Bureau [USCB], 2012), reaching 16.7% of the total population. This growth was expected to continue and researchers project that by 2060 Hispanics will make up 30% of the United States population, having increased from 55 to 119 million persons (Colby and Ortman, 2015), reaching minority majority status along the way (Colby and Ortman, 2015; Preuss et al., 2019).

As the Hispanic population continues to expand nationally, their presence in higher education has also been noted. For example, the National Center for Education Statistics [NCES] (2018) reported that in 2016, Hispanics made up 3.2 million, or 18.9%, of all undergraduate student enrollees. As the number of Hispanic students in higher education has increased, so has their share of all degrees earned. The number of associate degrees earned by Hispanic students increased by 10 percentage points (from 10 to 20 percent) between 2000–01 and 2015–16; the count of bachelor’s degrees earned has also doubled, increasing by 6 percentage points (from 6 to 13 percent) between 2000–01 and 2015–16 (National Center for Education Statistics [NCES], 2018).

An educated Latino community equates to greater economic opportunities for Hispanics and the greater society at large (Nora and Crisp, 2009). However, as Garza et al. (2019, p. 2) noted, “while we have experienced an increase in educational attainment, the growth in higher education completion rates has not kept up with the growth of the Hispanic population, which can have a negative effect on the future of the community as well as the future of our nation.” Within higher education, Hispanics are overrepresented in the attainment of associate degrees but have lower attainment of undergraduate or graduate degrees, particularly in STEM fields. According to the National Center for Science and Engineering Statistics, Hispanics are one of three racial and ethnic groups underrepresented in STEM (Linley and George-Jackson, 2013). NSF’s 2018 Science and Engineering Labor Force report states “Overall, Hispanics accounted for 6% of employment in S and E (science and engineering) occupations, which is lower than their share of the United States population age 21 and older (15%).” The statistics are even worse for females: in 2015, Hispanic women made up 1.8% of the United States S and E workforce, despite representing 7.5% of the United States residential population aged 21 or older (National Science Foundation [NSF], 2018). These circumstances led Sharkawy (2015) to characterize the limited presence of degreed Hispanics and members of other minority groups in the STEM workforce as “one of the most challenging problems for science education researchers and policymakers.”

The problem of underrepresentation of race/ethnic minorities in STEM is particularly evident at the doctoral level. This issue is magnified by the fact that in comparison with all other STEM doctoral students, completion rates of racial/ethnic minority students tend to be lower and attrition rates tend to be higher (Sowell et al., 2015). For example, time-to-degree, or the length of time it takes to complete the degree, is an issue of concern among a wide range of stakeholders in graduate education (Bell, 2010). According to a study of under-represented minority (URM) STEM students in 21 doctoral programs, Sowell et al. (2015) found that the median doctoral time-to-degree for Hispanic students was 64 months. In terms of attrition, one-half of engineering, mathematics, and physical sciences URM students who withdrew from their doctoral studies did so in 21 months (Sowell et al., 2015).

The attrition of underrepresented minority scientists in their journey from the dreams of youthful scientific exuberance to an impactful research career should alarm us all (Fadeyi et al., 2020). This poses an urgent threat to scientific innovation by missing out on diverse minds and talent, and simultaneously exposes several ugly cracks in the American developmental journey of a scientist (Stanford, 2020). The professoriate in particular plays a critical role in dispensing knowledge to URM STEM students through course instruction, advancing the field through research, and mentorship. Like all sectors of the workforce, the professoriate should resemble the country’s demographics. Unfortunately, this is far from the case in most STEM departments nationwide. As a result, talented faculty from URM groups remain a poorly tapped resource (Fadeyi et al., 2020). The poor representation of underrepresented minority (URM) faculty in academia also results in little to no role models for undergraduate students of color, which may negatively impact their aspirations, persistence, sense of belonging in undergraduate school, consideration of graduate school, or entrance into the STEM workforce (Jacobi, 1991).

### Barriers and Supports for Hispanic Students in STEM

A number of research endeavors have focused on understanding the barriers and supports to Hispanic engagement and retention in STEM degrees and careers. Studies have largely focused on factors located within the relationships students have with family and faculty, individual factors, and institutional factors. A closer examination of each factor demonstrates different aspects of the individual experience and suggests that for Hispanic students, multiple aspects are interwoven and determine whether the student will engage and persist in STEM studies and careers.

#### Relationships

Across multiple research studies there is evidence that the relationship Hispanic STEM students have are pivotal in entering and persisting in STEM. Families and faculty members are critical to engaging and supporting Hispanic students in STEM careers. The presence or absence of these supportive relationships has been found to be critical to many Hispanic STEM students.

##### Families

Family plays a central role in many Hispanics’ lives so it is unsurprising that the research found multiple ways in which families influence the career trajectory of their children. Family influence ranged from students being encouraged to persist when experiencing obstacles, to focus on their coursework and attend college, while also providing emotional and financial support (Banda, 2012; Peralta et al., 2013; Carrandi Molina, 2016). In rich qualitative interviews, Contreras Aguirre et al. (2020) collected data on how students found parental support to be crucial, particularly when they doubted their abilities in school. In a case study of ten undergraduate senior year Hispanic students enrolled in STEM majors, Contreras noted that “the encouragement and support parents provided to their daughters’ influence(d) their major choices and persistence in STEM fields” (p.137). Students voiced how parents had encouraged them to attend college, offered emotional support when they were stressed by their academic work, increased their self-confidence and belief in their ability to do the work, and shared their pride in their children. As one participant noted, “I think they’ve always been very supportive, they always said, whatever you want to do we’re going to support you… I know that whatever I do they are proud of me” (p.140).

##### Faculty

A further relationship variable found in the Contreras Aguirre et al. (2020) case-studies was the report by students of the importance of their relationships with faculty. Students indicated faculty provided them encouragement to continue onto graduate studies with them, to come to them with questions or requests for recommendations, provided interview tips, and motivated students to stay in difficult classes.

In a different case study research, Sangiago (2012) aimed to understand the unique experience of low-income Hispanic students in STEM at an HSI. Student responses included many who shared that they did not have an adult at the institution that they felt provided access to “support, social capital, or guidance” (p. 141). Those students who indicated positive relationships with faculty noted that the key benefits of those relationships were the provision by their mentor of “an extensive network, knowledge, and resources to connect them with high-impact programs and resources, such as summer research, academic support, and mentoring experiences” (p.142).

Faculty relationships can be even more important at the intense doctoral level of study. To prepare Hispanic STEM majors to enter doctoral programs, the Ronald E. McNair Post Baccalaureate Achievement Program has been successful with a program focused on faculty mentoring and academic preparation activities offered to students in their junior and senior years, providing additional evidence of the potential for faculty relationships to support Hispanic STEM graduate pathways (Fifolt et al., 2014).

### Institutional Factors

Studies on Hispanic success in STEM tend to focus most frequently on research concerning institutional and individual impacts that hinder or support minority student success in STEM. Institutional impacts look to understand the factors at universities that either promote or inhibit URM success in STEM. A number of researchers have looked across multiple studies to try to find consistent impact factors that could then guide universities in developing programs and actions that positively impact Hispanic participation in STEM.

In a review of 59 studies on what institutional factors could support academic success in Hispanic college students, Winterer et al. (2020) found eight factors that influence success, listed in order by those with the greatest number of studies supporting the factors. The factors identified were “peer interactions, cultural climate, advising, coursework articulation, academic integration, support services, asset-based factors, and outreach” (p. 8). Of these factors, peer interactions and individual student-based assets are less able to be influenced by the institution. However, the other six factors are largely within institutional control. Cultural climate includes the diversity of the faculty and students and the support and resources available for students from diverse backgrounds and identities. Advising was shown to have either a positive or negative impact based on how it was received by the student and based on a positive correlation with frequency and quality. Academic integration looked at the student involvement with external academic parts of the college, showing a positive correlation with student involvement in “study groups, learning communities, social contact with instructors, meeting with academic advisors, and academic conversations with instructors” (p.13). Students suggested that the availability of tutoring centers and computer labs and similar support services is additionally important to them. For community college students, coursework articulation that ensures students are able to transfer their credits to a 4-year institution, while important, did not come up in the studies as strongly impacting student success as the other elements.

In a second study that looked at summarizing findings from multiple articles, Martin et al. (2019) looked at 74 studies that considered how to improve pathways of success for Hispanic students. Positive outcomes were found for a number of institutional practices, including “mentoring, counseling, advising, study groups, tutoring, scholarships, orientations, career services, undergraduate research, articulation agreements, and transfer programs” (p. 3). However, the authors concluded none of the interventions had enough support to recommend wider adoption.

While the degree of support is uneven for the multiple interventions suggested in the reviews of the literature, the three areas advocated by the Hispanic Association of Colleges and Universities [HACU] (2020) task force have been frequently supported in research articles. The HACU formed a task force to create recommendations that would increase the participation and success of Hispanic students in STEM. For the community college and university level they recommended that there be “effective articulation programs, stronger laboratory STEM… (and) expanded undergraduate research opportunities” (p.13). Increasing articulation programs to keep 2-year STEM graduates from being discouraged or financially unable to complete a bachelor’s degree is essential and is supported in the literature (Jackson et al., 2013; Boatman and Soliz, 2018; Martin et al., 2019; Taylor, 2019; D’Amico et al., 2021).

Similarly, the recommendation to encourage institutions to use research and project-based experiences as a means to increase URM engagement in STEM has also been well-supported in the literature (Hackler, 2011; Slovacek et al., 2012; Foertsch, 2019; Jin et al., 2019; Ing et al., 2021).

### Individual Factors

A number of studies have focused on factors within the individual to explain Hispanic student retention and progress on a STEM pathway. Some of the newer research studies examining these individual-focused factors take an asset-based approach and suggest that Hispanic students possess traits, experiences, or abilities that support their STEM trajectories (Gallard et al., 2016). Other studies focus on more of a deficit approach such as inadequate academic preparation. Individual factors that have been studied include; number and level of science and math coursework (Wang, 2012; Borman et al., 2017), SAT and ACT math scores (Crisp et al., 2009), achievement in middle school and high school (Borrego et al., 2018), levels of self-efficacy (Wang, 2012; Borrego et al., 2018), persistence, networking, and race (Frett, 2018), gender stereotypes (Cunningham, 2017), exposure to math and science Wang, 2012), gender and ethnicity (Borrego et al., 2018), personality type, and genuine interest in the field.

A growing number of scholars critique much of the research and institutional approaches to URM student retention in STEM that uses a deficit model that locates the problem within the student rather than the institution (Harper, 2010; Valencia, 2010; Martin et al., 2019). The adoption of the Community Cultural Wealth model (Yosso, 2005) is an attempt to resituate how we conceptualize URM student success in higher education. In talking about cultural capital there are two distinct types discussed in the literature. Cultural capital is seen as that knowledge and experience that is generally available to majority members of the population with middle or high SES incomes. A newer form of cultural capital that is considered is Yosso’s (2005) Community Cultural Wealth (CCW) model. Yosso’s model focuses on “the forms of capital that draw on knowledge students of color bring with them from their homes and communities (Yosso, 2005, p.69).”

More recent studies have begun to use Yosso’s lens to consider how these types of knowledge and experiences found in URM communities provide resistance to oppression and resilience, allowing URMs to persist and thrive in STEM pathways. Drawing on two previous research studies, the authors Rincón and Rodriguez (2021) summarized the six forms of CCW as seen in Hispanic students pursuing STEM pathways. The forms of CCW they documented are; aspirational capital (hopes for STEM future), familial capital, linguistic capital, resistance capital (challenging inequality through oppositional behavior), and social capital (leveraging networks) thus providing support for Yosso’s theory of CCW.

In a phenomenological study that involved interviewing 16 STEM major Hispanic students, results further supported Yosso’s notion of the importance of Community Capital Wealth (CCW) (Rincón et al., 2020). In this research they found that second-generation college students had access to both traditional cultural capital and CCW, while first-generation college students only have access to CCW. The second- gen students spoke of the additional need to navigate between these two forms of capital.

In a third studying looking at CCW among URMs at a Primarily White Institution (PWI), Chavez (2018) found that “aspirational, familial, navigational, and resistant capital” (p. 141) were the most often used forms of Yosso’s CCW among her research participants. Students indicated that they used their navigational and resistant capital to deal with “instances in which participants described their response to a discouraging event or what their experience was like as a Hispanic STEM major, involved the feeling of being underprepared for STEM college courses, and the culture shock of attending a predominantly White university” (p 144). Students reported that their classmate(s) had come from well-resourced schools with AP courses in addition to having greater financial resources available to support themselves. Hispanic students used their CCW to deal with these challenges and were able to persevere and remain on a STEM pathway. The two approaches to cultural capital are useful as they both offer means of supporting students in STEM success.

### Next Research

Despite the many studies that have outlined the basic contours of barriers and supports for Hispanic participation and success in STEM, there remain calls for additional research. Crisp and Nora (2012) noted the need for deeper research into the “socio-cultural variables influencing Hispanic students’ decisions to major and persist in STEM (p.12).” Winterer et al. (2020) suggest that key barriers and supports have been clearly identified, however, there is a need for research that emphasizes how “institutional policies, practices, and programs” are experienced by individual students (p.20). Qualitative research with deep rich narratives is well suited to gain that greater degree of description of participant experiences.

## Background

The City College of New York (CUNY) and the University of Texas at El Paso (UTEP) were awarded a 5-year grant (H-AGEP) to develop a model program for preparing Hispanic STEM doctoral students to teach at 2-year colleges. The grant program includes three main components; teaching training, undergraduate mentoring, and workshops for professional development.

The teaching training program involves seven modules related to undergraduate STEM teaching and learning (CIRTL) which leads to Fellows creating a STEM course syllabus, lesson plan, teaching, philosophy, and diversity statements by the end of the semester. At the same time, Fellows participate in a practicum at a local community college where they are paired with a CC mentor from a similar background and major. The fellow initially observes their mentor but eventually teaches at least twice in that CC classroom, though some students have taught more frequently. The Fellows are provided continual feedback and mentoring from their CC mentor. Fellows also have a chance to work more closely with CC students by partnering with them to conduct research. Further, Fellows are provided a range of professional development workshops to support additional skills such as preparing for academic interviews and grant writing. Fellows also participate in evaluation and research activities as well as retreats and networking events throughout their participation in the H-AGEP program.

## Research Methods

The research being conducted as part of this NSF-funded grant seeks to understand how H-AGEP Fellows make career decisions, particularly to understand the paths these Fellows took to be in a STEM doctoral program, on the cusp of successful graduation. Social cognitive career theory (SCCT), developed by Lent et al. (1994), was the theoretical framework used in the development of semi-structured interview questions used to gather qualitative data about Fellow’s career decision-making. SCCT looks to explain three aspects of career decision making: (1) how individuals develop their academic and career interests; (2) how individuals make educational and career decisions; (3) how individuals attain academic and career success. This lens was useful in addressing the following research questions in our study:

Research Question 1: How did Fellows develop an initial interest in STEM?

Research Question 2: How did Fellows end up in STEM Ph.D. programs?

### Study Design

This study used a qualitative research design based on a grounded theory approach developed by Glaser and Strauss (1967), whereby researchers were interested in exploring a social construct from the perspective of the individuals being studied. In this research, we were particularly interested in learning about the career decision-making processes of Hispanic STEM doctoral students and how these influence their decisions to pursue academic careers. The findings generated seek to add rich descriptions of participants experiences and perceptions of their experiences.

The NSF’s Alliances for Graduate Education and the Professoriate (AGEP) program contributes to the National Science Foundation’s objective to foster the growth of a more capable and diverse research workforce (National Science Foundation [NSF], 2018). Through this solicitation, the NSF seeks to build on prior AGEP work, and other research and literature concerning racial and ethnic equity, to address the AGEP program goal to increase the number of historically underrepresented minority faculty in STEM. Furthering the AGEP goal requires advancing knowledge about new academic STEM career pathway models (Alliances for Graduate Education and the Professoriate [AGEP], 2021). The use of the term “historically underrepresented minority” reflects language from Congress, and in the context of the AGEP program, the AGEP populations are defined as STEM doctoral candidates, postdoctoral scholars, and faculty who are African Americans, Hispanic Americans, American Indians, Alaska Natives, Native Hawaiians, and Native Pacific Islanders. At the graduate student level, only doctoral candidates are included because they have greater potential to enter a faculty position within the project duration time frame. Therefore, in terms of participant recruitment for this study, a non-probabilistic, purposive sampling approach was used, as only H-AGEP participants were selected according to predetermined criteria (Hispanic STEM doctoral students) relevant to our research objective related to the analysis of career decision-making.

### Participants

Participants in this study consisted of thirteen Hispanic STEM doctoral students from both the first and second cohort of the H-AGEP NSF-funded grant. The demographic characteristics of these participants, referred to as H-AGEP Fellows, are shown in Table 1.

**TABLE 1:** Demographic characteristics of H-AGEP fellows (*N* = 13).

Gender	Hispanic Sub-Group	1st Gen/2nd Gen	Major
Male	Mexican	2nd Gen	Civil Engineering
Male	Mexican	2nd Gen	Electrical Engineering
Male	Colombian	2nd Gen	Electrical Engineering
Male	Colombian	2nd Gen	Mechanical Engineering
Male	Ecuadorian	1st Gen	Environmental Sciences
Male	Puerto Rican	2nd Gen	Mechanical Engineering
Female	Peruvian	2nd Gen	Biology
Female	Puerto Rican	2nd Gen	Oceanographic Sciences
Female	Mexican	2nd Gen	Environmental Sciences
Female	Mexican	2nd Gen	Biology
Female	Mexican	1st Gen	Biology
Female	Mexican	1st Gen	Ecology/Biology
Female	Mexican	1st Gen	Civil Engineering

Participants represent a range of Hispanic subgroup identities. Seven H-AGEP Fellows are from Mexican backgrounds and the other six are from Colombia (2), Ecuador (1), Peru (1), and Puerto Rico (2). A third of the Fellows (4) attended non- United States institutions as undergraduates. Of the nine who attended United States institutions, four received Pell grants and five did not. Six of these students had attended a 2-year college at some point in their academic journey. Most students were not first-generation college students (9), though four were. Students represented a range of STEM majors with a heavier emphasis in engineering (Electrical Engineering-2, Mechanical Engineering- 2, Civil Engineering-2, Environmental Science and Engineering-1), followed by Earth and Environment majors (Earth and Oceanographic Sciences -1, Environmental sciences -2, and Ecology and Evolutionary Biology-1) in addition to three students majoring in Biological Sciences.

Fellows were initially recruited to this grant by H-AGEP Alliance members. These grant team members are faculty at CCNY and UTEP who teach in STEM fields. To qualify to be a Fellow, Hispanic STEM students had to have completed all of their doctoral coursework, be at the dissertating stage, have advisor permission, and have an openness to completing the components of the program. At the time interviews were conducted, these Hispanic doctoral STEM students were in the mid-phase of their program, where most had taken their comprehensive exams and had, or were in the process of, defending their dissertation proposal.

### Interviews

Prior to initiating interviews with the H-AGEP Fellows, the lead researcher developed interview questions using a SCCT lens that sought to elicit aspects of Fellow’s career decision-making processes. Interview questions were centered around six key areas thought to influence career decision-making: (a) the development of STEM interest at home and at school; (b) interactions within K-12 and higher education systems around their STEM interest and developing STEM identity; (c) experiences in their doctoral program; (d) impacts of Hispanic and gender identities; (e) influences of geographic mobility; and (f) Fellow’s experiences in the H-AGEP program. Interview questions were semi-structured to allow for both formal and informal discussions about each topic or to expand onto a related topic.

H-AGEP Fellows were contacted by the Graduate Student Research Assistant in late 2019 to inform them of the purpose of the study and to obtain their written consent for participation. Students were assured that participation was voluntary, confidential, and that they could choose to stop the interview at any time. IRB approval was granted from the lead author’s institution (University of Massachusetts at Amherst) as well as from the partner institutions (UTEP and CCNY). Interviews were conducted by the Research Lead and the Research Assistant in January and February 2020 either in person or by the Zoom online platform depending on convenience for the student. A semi-structured interview protocol was used to ensure that all students were asked the same questions, though Fellows were able to digress from the questions and add material not requested. Interviews lasted anywhere from 50 to 90 min and were recorded and then transcribed for accuracy.

### Data Analysis

Interviews were recorded on the Zoom platform and later transcribed by Rev.com. Once the transcriptions were completed, data was downloaded onto the MAXQDA qualitative and mixed methods data analysis software program. A grounded theory methodological approach was used to identify themes related to Fellow’s development and support of their STEM interests and how these experiences affected their decision to pursue a Ph.D. in STEM.

Grounded theory refers to a set of systematic inductive methods for conducting qualitative research aimed toward theory development (Given, 2008). A grounded theory approach was chosen because it (a) provides explicit, sequential guidelines for conducting qualitative research; (b) offers specific strategies for handling the analytic phases of inquiry; (c) streamlines and integrates data collection and analysis; (d) advances conceptual analysis of qualitative data; and (e) legitimizes qualitative research as scientific inquiry (Given, 2008).

Data analysis was an iterative process whereby each researcher collected and analyzed the interview data and used inductive reasoning to create codes as each transcript was read. For each interview, the researchers recorded new codes developed and noted code characteristics, including the code name, code definition, type of code (inductive or deductive), any notes about the new code (e.g., clarity of the issue, completeness of the code definition). New codes were generated and grouped within the following categories: development of STEM interests in k-12 schools, development of STEM interests outside of school, undergraduate experiences, pursuit of graduate degree, mentorship experiences, impacts of gender and/or ethnic identity, and H-AGEP experiences. Documentation of code development and iterative refinement of codes continued for each interview individually until all thirteen interviews were reviewed and the codebook was complete. Areas of disagreement were discussed and reconciled so that there was agreement on all codes and coding. After two transcripts there was sufficient consistency for the two coders to work independently. As new codes emerged the coders met to discuss and work backward to see if they applied to any previously coded transcripts. After all the transcripts were coded, a review of the codes was pursued to develop initial themes which were used to answer our research questions.

Using a grounded theory approach, conducting a rigorous data analysis generally results in the identification of all available codes relevant to the research inquiry. Achieving this end point is often referred to as saturation, where no additional issues or insights emerge from the data and all relevant conceptual themes have been identified, explored, and exhausted (Hennink et al., 2017). This signals that conceptual categories are “saturated,” and the emerging theme, or theory, is comprehensive and credible. Sample sizes recommended for qualitative research vary, but previous studies have found that saturation, based on the extent of theme development and theme importance in the data, can be achieved at twelve interviews (Guest et al., 2006; Hennink et al., 2017). Therefore, our dataset of thirteen interviews was considered sufficient for pursuing our research objectives.

## Results

Results were analyzed to see what themes developed surrounding each of the research questions. In response to the first research question, *how did Fellows develop an initial interest in STEM*, we found five distinct areas of influence in how Fellows developed their initial interest in STEM. The respondents overwhelmingly indicated interest development at an early age. The key areas noted by participants included: the influence of parents as teachers, engagement with family construction companies, the outdoors, access to taking things apart, and access to books. Through these varied activities we found that the Fellows developed a passion for STEM.

### Parents as Teachers

A number of Fellows discussed how their parents acted as teachers and increased their knowledge, skill, and interest in STEM. However, these parents were not acting in traditional teaching with lecture modes with large groups of students but instead were engaging their children in hands-on science or individualized tutoring in mathematics.

• One Fellow noted, “My mom, she’s actually very good at it, and she was the one.” You know like when you have the breaks, she was always like, okay, let’s go further. Let’s take it a little bit. So, she was teaching me (math) so that when I was going to the next year, I was ready. And I never had an issue, never fought with her because of that. I liked it.
• Since I was little, my mom’s a science teacher. So, I grew up with science lessons if I wanted to or not. So, I grew up with learning science and doing science projects always. For me it was doing experiments at home, so she would bring stuff that she would teach in the class and then do it at home with me. And, when I was younger, I would go to class with her. So, I grew up dissecting stuff in her classroom.

### Construction Companies

Other Fellows discussed going to work with parents or other family members who owned or were involved in construction companies. Through their family connections they built informal networks that provided access to experiences others without those connections would not have. Authentic engineering experiences in the world intrigued our students as they participated in these family business activities.

• One of my uncles is an electrical engineer and my dad is an architect. He had a construction company, and so since I was a little kid, I was around them. I always liked that. I liked building and doing… we would always be doing projects at home. We had a pretty big house and land, so we were always building stuff there. Then with my uncle, we would do some electrical work, but that was when I was older, in middle school. That’s where I got shocked few times, helping there.
• Before high school. I have an uncle, well, it’s not really a direct uncle, but he’s part of the family and he has a business. He sells materials for construction. And I think that was something that when I talked to him, I got interested in civil engineering especially…, when I was in high school, I had to do some service hours, so I decided to do my hours with my uncle. I was able to go to different construction sites and talk to people at different projects and talk to those guys and see how they were doing it, and what is what they were doing. And so, I was able to go to their field and interact with people and talk to them… and just from that experience I was like, “Oh yeah, I think this is something that I enjoy and I like.”

### Outdoors

Other Fellows were drawn into STEM through their experiences with the outdoors, an opportunity provided by their families. The Fellows’ experiences in nature varied but they all spoke of how this had connected them to STEM in a deep way.

• When I was a child, I always used to go to my grandparents’ farm. They have a pair of forests and pair of farms there. That’s the way I remember I like nature. I always see how life moved when I was there. So, I was interested how this happens, why this happens. This was at age 6–8 years old.
• So, I actually always loved being in nature. I think the thing is, my parents always loved to be outdoors. We’ll go camping or having a picnic in the river. And in (country name redacted), it’s very easy to go just from the beach to the mountains, so it was always at that time when I was young, I was very privileged. We had the opportunity to travel also to the countryside. And then I think when I was in school, I remember I really loved my natural science class. And I remember when I was a kid, I wanted to be maybe a geographer or, I always dreamed about traveling to new places. And being like an explorer, right?
• My family is really outdoorsy. We go camping a lot. Go hiking a lot. We were always looking at animals and plants and rocks and stuff like that. It was just really having a childhood where we were outdoors a lot and going hiking and going all over the place.

### Taking Things Apart

For some Fellows their interest was sparked by curiosity about how things worked and the occasional opportunity to pull things apart and see. Families that provided these opportunities to their children stimulated and supported their child’s deep interest in STEM.

• So, I liked to be fixing things, moving things, see how they work when I just take apart some equipment and put it back together.
• Maybe when I was 10 years old, and I had my first video game console and it got me curious about electronics… a curiosity of understanding how they worked.

### Books

For some Fellows, literature was the key introduction to a love for STEM. Through the pages of books provided by parents they became drawn into STEM. Parents were also seen as instrumental in providing additional materials and experiences stimulated with literature.

• I was very young, I would say. So, even in elementary school, I guess. I was interested in like space… Kind of always had a fascination with it (since) third grade. I think I did read children’s books but space oriented. And then I feel like my parents would try. (Parents took her to space observatory and Cape Canaveral as child).
• I wanted to do airplanes. I remember it developed because my parents had. if you remember the almanacs, if you remember those books, my parents had that and I remember looking, just flipping through one of them and I saw a picture of helicopter and that’s where I remember that first bird. And I remember I turned the pages very slowly in that section. And so that’s where my interests were, and then I didn’t really pursue kind of at that time the whole idea of STEM, it was not in my verbiage. But I knew I wanted to do something with science and math, I knew I liked it.

### Varied

A few of our students had unique triggers for the passion for STEM. In one case it was their AP classes, in another their love of bridges, and lastly a family illness. These triggers had the same impact as the previously listed categories in that Fellows reported these events was where their desire to pursue STEM originated.

• HS CLASSES: It was the classes that I was taking. So, I had the AP courses, so all the biology (*sic*) and chemistry courses were a little advanced so I that’s why I wanted to learn it because I really enjoyed it more than math. And… all of the science fairs, I really liked the science fairs.
• BRIDGES As a child (I was) in love with bridges. Later realized that meant engineering and since (I) liked math (I) wanted to do something with bridges.
• FAMILY ILLNESS… (my) grandma got diagnosed with breast cancer when I was very young and that changed my perspective on medicine. So that’s when I wanted to become a doctor.

Both researchers noted that when the interviewees discussed their pathways to STEM, we heard a passion for STEM. Students appeared emotionally motivated by the intrinsic joy they received from engaging with STEM. These early life activities appear to have ignited an interest in STEM that has continued into their doctoral programs. In discussing their academic choices and journey, students all seemed to assume they would pursue a bachelor degree in STEM. They did not hold the same assumption, however, when the transition was to doctoral studies.

Research question two, *how did Fellows end up in STEM Ph.D. programs*, provided insight into how Hispanic students with a demonstrated interest in STEM described their individual journeys from a student with a passion for STEM to being doctoral candidates in STEM. Five emergent themes that influenced their pursuit of a Ph.D. included; chance encounters with peers, issues of employment, interactions with professors, coursework, and self-initiation.

### Chance Encounters With Peers

More advanced peers in their doctoral program provided Fellows with cultural capital through the information they provided about the doctoral process or which professors to seek out. Peers who were similar in age or ethnicity to the Fellow were particularly valued.

• One of the reasons I decided to do a Ph.D. is because when I joined this lab as a graduate student, my advisor had a Ph.D. student who was Peruvian so we kind of had a good connection right there. A doctorate honestly back then sounded out of reach for me, I hadn’t even considered it but after meeting this guy, I kind of identify (with him) and basically, I thought that I could do it as well because I saw a lot of similarities. He did his undergrad back home, he came here (to the United States) for grad school, he’s been in the lab so having that connection with him and also having similar backgrounds pretty much brought the idea closer to me, into consideration.
• And when I would go to get help with homework from his TA, who was a Chinese student, an international student, I was asking him about the Ph.D. and things like that, and it was like, “Oh wait, you’re getting paid to do this? You’re getting your tuition taken care of?” And then I literally asked him, “so you’re saying that I can say that I want to do a Ph.D. and I don’t want to pay for it and I could be arrogant about it?” And he’s like, “yeah, yeah, you can, you can get paid to go to school.” I’m like, “what?”
• So, I didn’t interact with anyone, not even the professors, but one professor, I think it was almost when I was finishing my senior year (of the undergraduate), Dr B. my advisor, well one friend recommended me to go talk to him to start doing research. I talked to him and he offered me (a research position) to start as a volunteer. And again, everything changed after that because I got more involved.

In one case a student finds that seeing another Hispanic student doing doctoral work provided him with the self-confidence to recognize he is also capable of doing that. Another student found that doctoral studies would not be the financial struggle they envisioned and was thus something they could consider. And lastly, through a peer pointing them to a professor that would provide research opportunities for undergraduates, a student became involved with a professor who would further support him into pursuing doctoral studies.

### Employment

For some students, transitional points in employment offered them the opportunity to consider applying to school. Students at these transitional points seemed equally happy to continue working or return to school to obtain a masters and doctoral degree-.

• The engineering students here take an exam at the end of their careers called the FE from the… engineering. So, my first step was to sign up for that exam… so I did (the) exam, I passed and my plan was after the exam, look for job or apply to school. And I was working, so in the meantime I was applying for school for the masters and I waited until I had my certification from the exam to apply for engineering jobs. But I guess maybe since I started the application process for school earlier maybe I got a response sooner. So, I was testing my options and I guess I got the response from the graduate schools first before I was able to find a job.
• That’s a little. I guess it was. I didn’t plan for it. Honestly, I never thought of. I mean, I maybe had slight idea of applying for a (graduate degree). It wasn’t in my first option. But then I was working in (foreign country) for a company, then they had some changes there… my contract was up and they told me that I have to wait to renew the contract. Again, I had thought about the Ph.D., and then at that point I was like, “You know what, I’m thinking this might be a good opportunity to pursue it,” and then I applied, and I got accepted.

### Professors

Professors were key to some students in the networking they provided, and in others, in the critical cultural information about the Ph.D. process that was provided. In the first example, the seamless transition from one advisor to another at a different institution was facilitated by the networking of the first professor for her students. In the second example a student gained critical knowledge, that while not deployed immediately, in the end served to lead the student to entering a STEM doctoral program.

• My undergrad advisor knew my current advisor M. And so, she knew she was looking for students, so that’s kind of how. And then I stayed with M through the Ph.D. program.
• There was a professor here, her name was Dr. X.… But at that point in time I didn’t know what to do (at end of BA) and since my parents didn’t go to college, I didn’t know that there was anything other than a bachelor’s degree. So, she was really nice and she took an hour and a half just explaining to me that there is a Master’s program, there’s a Ph.D. program. I later took up a job at (retail job), and I was up for a promotion because I was the only employee who had six of six standards. And when I got my promotion, it was 65 cents so I said, “You guys can’t just add 65 cents.” The very next day I called in sick, I applied… I came here, I found out I only had a week to turn everything in (for Ph.D. application). But I wouldn’t have known about a Ph.D. program if it wasn’t for Dr. X, and that’s because she took the time to tell me. In my science courses there was never people saying, “There’s much more than just a bachelor’s degree, you can do science research, or you can do this, or you can do that.”

### Coursework

For one student, an exciting experience in a community college biology class engaged them so much with biology that from that experience, they started on the path to the Ph.D.

• Taking a class at CC. I took my intro to biology courses there. Which I had put off, because I didn’t like. I almost failed biology in high school. And I really hated it. That’s one of the reasons why I took biology at CC, because they said it was easier. And I had a really good professor that made me like biology. For sure taking that course with that professor that I had at CC (led to entering doctoral program). Because I thought to myself, “If only my high school teacher was like that, then maybe I would have decided a long time ago that this is what I wanted to do. And I wouldn’t have wasted so much time doing other stuff.”

### Self-Initiated

A number of students indicated that they were their own motivator to pursue a Ph.D. One student decided that his home country did not offer the resources to pursue the type of doctoral work he was interested in. A second student indicated their desire to learn propelled them back into academia.

• When I was getting my undergrad, I got involved with some research. But we didn’t have enough instruments so that we can fully do research. And that’s when I decided to relocate to the United States I always wanted to go for higher education, but at that point I wanted to go for a Master’s. I actually wanted research, and I knew that going toward higher education probably will get me to that point. And then I came to the United States, and I got into a master’s program. While I was doing the master’s program, I was actually able to do research (that led to pursuing the Ph.D.).
• I moved to NYC and after completing my undergraduate degree and I decided to apply for a graduate degree. I was not pushed by a person. I would describe it as, “Because I like to learn more things.”

### Additional Finding

While this study looked at critical incidents on the path to the STEM doctorate, we also found an unexpected finding that may provide further corroboration of the Sowell et al. (2015) study results. As of year four, all of the Fellows are persisting toward their doctoral degree or have graduated. Three of the four critical factors that were found as central to URM student persistence to degree are central parts of this grant program; financial support, mentoring/advising, and peer group support.

Students are provided a stipend for participating in the program and additional funds when they participate in the teaching practicum in a partnering community college. Additional funds are made available for those Fellows who provide research mentoring to undergraduates and for conference travel expenses.

The development of a strong mentorship and networking program to support Fellows during their transition to academic careers in community colleges and in their professional lives is another key pillar of this intervention. Mentorship is provided by grant faculty, the Fellow’s dissertation advisors and community college faculty. All work in concert to ensure that the Fellows are supported in gaining the skills needed for transitioning successfully to a career and while in the program.

Finally, Fellows often described the social support they received from the frequent interactions with their peers within and across the two lead institutions. The social support and extended peer network developed over the course of the program—as Fellows graduated and a new cohort was welcomed—provided Fellows with role models of successful URM STEM peers that they may not have otherwise seen in their programs or at their institutions. The connections established between the Fellows reinforced to them the similarities in the challenges URM STEM doctoral students face as they contemplate pursuing academic careers, while also reinforcing the values these Hispanic Fellows prioritized in being role models for the next generation of Hispanic, URM undergraduates.

## Discussion

Research has shown that Hispanic students are interested in STEM majors and careers at the same level as White students. However, many fewer Hispanics end up graduating with STEM degrees and this is especially so at the doctoral level where only three percent of STEM doctorates are awarded to Hispanics (U.S. Department of Education [USDE], 2012). It raises the obvious question of what happens between the development of interest in a STEM major and degree attainment. This research is unique in looking in particular at the varied pathways to the doctorate in STEM. In reviewing the data, it appears that while there are commonalities in how the Hispanic students surveyed developed their STEM interests, there was a wide range of non-structured, random events leading to Hispanic STEM student participation in doctoral programs.

Consistent with prior researchers (Banda, 2012; Peralta et al., 2013; Carrandi Molina, 2016), we found that family support was instrumental in students engaging and continuing in STEM pathways. Similarly, we found agreement with Contreras Aguirre et al.’s (2020) work that found that positive relationships with faculty could similarly support students on their STEM pathway. While prior research studies have indicated the importance of faculty mentoring and contact with instructors (Martin et al., 2019; Winterer et al., 2020) and we found that was an important factor for some of our Fellows, the presence of such a supportive faculty member was inconsistently present for our students and instead they more often relied on families, peers, and the cultural capital (or CCW) they brought with them to succeed.

While family support and CCW were critical to participants developing a passion for STEM at a young age and leading to student enrollment in STEM baccalaureate programs, the CCW they developed then did not include gains in CCW that would support entry into doctoral programs. As a result, while all our students spoke of their early goals of pursuing STEM bachelor’s degrees, the doctorate was not assumed by our Fellows. The decision to pursue a doctorate seemed to require the majority of students to engage with faculty or peers in such a way that then the path opened up to them. In looking at this doctoral engagement process, we found that there seemed to be critical chance incidents that occurred and ended with students entering doctoral programs. We define *critical incidents* as something that happens that leads to a bifurcation in the direction of the Fellow’s career path, and with them pursuing a path that was less likely or not available prior to the critical event.

• Example: Professor X met with a Fellow for 90 min to explain the structure of advanced study (BA - > Masters- > Ph.D.) Later, this knowledge allowed the Fellow to pursue the path to the Ph.D.

Further, these critical incidents often occurred by chance and were not planned or part of a structured event. These were not events experienced by all undergraduate STEM majors.

• Example: A number of Fellows by chance were in labs when a peer, with critical knowledge or serving as a role model, was present.

The probability of the occurrence of these critical events, that led our Fellows to doctoral programs, are a concern as there is a strong element of uncertainty to these chance events. This leads to the question of how many more Hispanic students might have continued on the path to a doctorate if they had experienced these types of events. It also suggests that the critical cultural knowledge that was represented in these events may be essential to disseminate to all Hispanic STEM undergraduates and other students underrepresented in STEM. Institutions that wish to increase Hispanics in STEM graduate work may want to consider how to institutionalize the sharing of cultural capital about STEM pathways in programmatic rather than provisional ways.

These research results do not appear to support the idea seen in many K-12 interventions that STEM programs, offered in varied amounts across school systems and within local communities, are the primary catalyst for students developing a passion for STEM. Instead, we found that families that respond to their child’s interests and support that development is what was effective with our participants in putting them on a direct path to STEM careers.

Future research is needed to understand the parenting practices that have so frequently been seen in our Hispanic Fellows’ experiences. Is it possible to share those practices within different types of URM communities? Additionally, research on creating effective interventions that explore sharing key cultural knowledge about the graduate school pathways to STEM with Hispanic undergraduates is also desirable. This research demonstrates that, as others have found, cultural capital is of great importance in Hispanic student academic engagement. We did not collect data on CCW but it would be worthwhile in the future to see how CCW provides the cultural capital beneficial for student success beyond attainment of the bachelor’s degree.

### Limitations

As with any case study our findings are limited by the small size of our sample. Our findings cannot be generalized to other Hispanic doctoral STEM students or other URM students pursuing doctoral degrees. We will be repeating this study with the next two cohorts and look to see whether future data supports our initial findings or whether new themes are identified that influence career decision-making. Additionally, we may not be able to draw conclusions about the STEM pathways for all Hispanic students, particularly for those that do not have and will never have an interest in graduate studies. There may be differences in motivation and curiosity, that our Fellows showed an abundance of, and that make them more likely to wish to engage in doctoral studies where they can continue to think and explore ideas and questions. However, identifying children with a passion for STEM may be a useful first step in finding and supporting future STEM college graduates.

## Conclusion

This research has found that Hispanic families and the manner in which they engaged their children in STEM activities or supported their child’s interests was uniquely effective in leading to doctoral Fellows with self-motivation to pursue their passion—STEM. Additionally, the importance of critical cultural knowledge about the academic process of graduate school should not be assumed to be known or available to many Hispanic undergraduate students. Without this knowledge, even Hispanic students who have a passion for STEM may be lost to engagement in graduate STEM pathways.

## Data Availability Statement

The datasets presented in this article are not readily available due to restrictions in the submitted IRB. Requests to access the dataset should be directed to DH, dmhorton@umass.edu.

## Ethics Statement

The studies involving human participants were reviewed and approved in an IRB submitted at the University of Massachusetts, Amherst. The patients/participants provided their written informed consent to participate in this study.

## Author Contributions

DH developed the interview protocol with suggestions and revisions by IT-C. DH applied for and received IRB approval. DH and IT-C each conducted half of the interviews and each coded 3/4 of the interviews in a qualitative data software. DH themed the results and reviewed with IT-C for suggestions and revisions. DH wrote the article with input from IT-C. Both authors contributed to the article and approved the submitted version.

## Conflict of Interest

The authors declare that the research was conducted in the absence of any commercial or financial relationships that could be construed as a potential conflict of interest.

## Publisher’s Note

All claims expressed in this article are solely those of the authors and do not necessarily represent those of their affiliated organizations, or those of the publisher, the editors and the reviewers. Any product that may be evaluated in this article, or claim that may be made by its manufacturer, is not guaranteed or endorsed by the publisher.

## References

[B1] Alliances for Graduate Education and the Professoriate [AGEP] (2021). *Program Solicitation NSF 21-576.* Alexandria: National Science Foundation.

[B2] Banda R. M. (2012). *Perceptions of Social Support Networks and Climate in the Persistence of Latinas Pursuing an Undergraduate Engineering Degree.* Ph.D. thesis. Ann Arbor: Texas A&M University.

[B3] Bell N. (2010). *Data sources: Time-to-Degree for Doctoral Recipients.* Albany: CGS Communicator.

[B4] Boatman A. Soliz A. (2018). Statewide transfer policies and community college student success. *Educ. Finance Policy* 13 449–483.

[B5] Borman T. Margolin J. Garland M. Rapaport A. Park S. J. LiCalsi C. (2017). *Associations Between Predictive Indicators and Postsecondary Science, Technology, Engineering, and Math Success among Hispanic Students in Texas. REL 2018-279.* Washington: U.S. Department of Education.

[B6] Borrego M. Knight D. B. Gibbs K. Jr. Crede E. (2018). Pursuing graduate study: factors underlying undergraduate engineering students’ decisions. *J. Eng. Educ.* 107 140–163. 10.1002/jee.20185

[B7] Carrandi Molina E. (2016). *First-Generation Hispanic Freshmen Perceptions of Selecting a STEM Major.* Ph.D. thesis. Miami Shores: Barry University.

[B8] Chavez M. (2018). *Examining the Experiences of Latinx STEM Baccalaureates.* Ph.D. thesis. Chicago: Loyola University.

[B9] Colby L. Ortman S. (2015). *Projections of the Size and Composition of the U.S. Population: 2014 to 2060. Population Estimates and Projections. Current Population Reports.* Washington: U.S. Census Bureau, P25–1143.

[B10] Contreras Aguirre H. C. Gonzalez E. Banda R. M. (2020). Latina college students’ experiences in STEM at Hispanic-Serving Institutions: framed within Latino critical race theory. *Int. J. Qual. Stud. Educ.* 33 810–823. 10.1080/09518398.2020.1751894

[B11] Crisp G. Nora A. (2012). *Overview of Hispanics in Science, Mathematics, Engineering, and Technology (STEM):k-16 Representation, Preparation, and Participation.* Washington: Hispanic Association of Colleges and Universities.

[B12] Crisp G. Nora A. Taggart A. (2009). Student characteristics, pre-college, college, and environmental factors as predictors of majoring in and earning a STEM Degree: an analysis of students attending a Hispanic Serving Institution. *Am. Educ. Res. J.* 46 924–942. 10.3102/0002831209349460

[B13] Cunningham N. M. (2017). *STEM Success: Perceptions of Women of Color at Community Colleges.* Ph.D. thesis. Scottsdale: Northcentral University.

[B14] D’Amico M. M. Chapman L. M. Robertson S. (2021). Associate in applied science transfer and articulation: an issue of access and equity. *Commun. Coll. J. Res. Pract.* 45 378–383. 10.1080/10668926.2020.1741477

[B15] Fadeyi O. O. Heffern M. C. Sanders Johnson S. Townsend S. D. (2020). What Comes next? simple practices to improve diversity in science. *ACS Cent. Sci.* 6 1231–1240. 10.1021/acscentsci.0c00905 32875063 PMC7453409

[B16] Fifolt M. Engler J. Abbott G. (2014). Bridging STEM professions for McNair scholars through faculty mentoring and academic preparation. *Coll. Univ.* 89 24–33.

[B17] Foertsch J. (2019). Impacts of undergraduate research programs focused on underrepresented minorities: twenty years of gradual progress and practices that contributed to it. *Scholarsh. Pract. Undergrad. Res.* 3 31–37.

[B18] Frett J. (2018). *College-to-Career Experience: Black and Hispanic First-Generation College Graduates.* Ph.D. thesis. Philadelphia: University of Pennsylvannia.

[B19] Gallard A. J. Pitts W. B. Claeys L. Flores B. B. Brkich K. Stevenson A. (2016). *Resiliency: A Unique and Dynamic Form of Cultural Engagement.* Washington: American Educational Research Association, 1–9.

[B20] Garza L. Pacheco G. Jr. Gallardo J. Castillo Y. Henderson E. (2019). Transforming the Hispanic leader. *J. Lat. Educ.* 18 427–433. 10.17226/10885 25009857

[B21] Given L. M. (ed.) (2008). *The Sage Encyclopedia of Qualitative Research Methods.* Thousand Oaks: Sage publications.

[B22] Glaser B. Strauss A. (1967). *The Discovery of Grounded Theory: Strategies for Qualitative Research.* New York: Aldine Publishing Company.

[B23] Guest G. Bunce A. Johnson L. (2006). How many interviews are enough? An experiment with data saturation and variability. *Field methods* 18 59–82.

[B24] Hackler A. S. (2011). *Redefining Science, Technology, Engineering, and Mathematics (STEM) Educational Opportunities for Underserved and Underrepresented Students at NASA.* Ph.D. thesis. Alabama: University of Alabama.

[B25] Harper S. R. (2010). An anti-deficit achievement framework for research on students of color in STEM. *New Dir. Inst. Res.* 148 63–74. 10.1002/ir.362

[B26] Hennink M. M. Kaiser B. N. Marconi V. C. (2017). Code saturation versus meaning saturation: how many interviews are enough? *Qual. Health Res.* 27 591–608. 10.1177/1049732316665344 27670770 PMC9359070

[B27] Hispanic Association of Colleges and Universities [HACU] (2020). *Rethinking Policy and Practice for STEM Education: New Hispanic Perspectives.* San Antonio: Hispanic Association of Colleges and Universities.

[B28] Ing M. Burnette J. M. III. Azzam T. Wessler S. R. (2021). Participation in a course-based undergraduate research experience results in higher grades in the companion lecture course. *Educ. Res.* 50 205–214. 10.3102/0013189X20968097

[B29] Jackson D. L. Starobin S. S. Laanan F. S. (2013). The shared experiences: facilitating successful transfer of women and underrepresented minorities in STEM fields. *New Dir. High. Educ.* 162 69–76. 10.1002/he.20058

[B30] Jacobi M. (1991). Mentoring and undergraduate academic success: A literature review. *Educ. Res.* 61 505–532. 10.3102/00346543061004505

[B31] Jin L. Doser D. Lougheed V. Walsh E. J. Hamdan L. Zarei M. (2019). Experiential learning and close mentoring improve recruitment and retention in the undergraduate environmental science program at an Hispanic-Serving Institution. *J. Geosci. Educ.* 67 384–399. 10.1080/10899995.2019.1646072

[B32] Linley J. L. George-Jackson C. E. (2013). Addressing underrepresentation in STEM fields through undergraduate interventions. *New Dir. Stud. Serv.* 144 97–102. 10.1080/09553002.2021.1988182 34634981 PMC9676100

[B33] Lent R. Brown S. Hackett G. (1994). Toward a unifying social cognitive theory of career and academic interest, choice, and performance. *J. Vocat. Behav.* 45, 79–122. 10.1006/jvbe.1994.1027

[B34] Martin J. P. Choe N. H. Halter J. Foster M. Froyd J. Borrego M. (2019). Interventions supporting baccalaureate achievement of Latinx STEM students matriculating at 2-year institutions: a systematic review. *J. Res. Sci. Teach.* 56 440–464. 10.1002/tea.21485

[B35] National Center for Education Statistics [NCES] (2018). *Fast facts: Enrollment.* Available Online at: https://nces.ed.gov/fastfacts/display.asp?id=98 (accessed May 21, 2021).

[B36] National Science Foundation [NSF] (2018). *Building the Future: Investing in Discovery and Innovation.* Available Online at: https://www.nsf.gov/publications/pub_summ.jsp?ods_key=nsf18045 (accessed May 28, 2021).

[B37] Nora A. Crisp G. (2009). “Hispanics and higher education: an overview of research, theory, and practice,” in *Higher Education: Handbook of Theory of Research*, ed. Smart J. C. (Berlin: Springer), 321–358.

[B38] Peralta C. Caspary M. Boothe D. (2013). Success factors impacting Latina/o persistence in higher education leading to STEM opportunities. *Cult. Stud. Sci. Educ.* 8 905–918. 10.1007/s11422-013-9520-9

[B39] Preuss M. D. Rodin J. C. Sosa E. M. Ramos J. D. Dorsett C. R. Burleson C. R. (2019). *Hispanic-Serving Institutions in the South-Central United States: A Research Report for Los Barrios de Amarillo.* Alexandria: National Science Foundation.

[B40] Rincón B. E. Rodriguez S. (2021). Latinx students charting their own stem pathways: how community cultural wealth informs their STEM identities. *J. Hispanic High. Educ.* 20 149–163. 10.1177/1538192720968276

[B41] Rincón B. E. Fernández É Dueñas M. C. (2020). Anchoring comunidad: how first- and continuing-generation Latinx students in STEM engage community cultural wealth. *Int. J. Qual. Stud. Educ.* 33 840–854. 10.1080/09518398.2020.1735567

[B42] Sangiago C. (2012). *Faculty as Institutional Agents for Low-Income Latino Students in Science, Technology, Engineering, and Mathematics Fields.* Ed.D. dissertation. Los Angeles, CA: University of Southern California.

[B43] Sharkawy A. (2015). Envisioning a career in science, technology, engineering and mathematics: some challenges and possibilities. *Cult. Stud. Sci. Educ.* 10 657–664.

[B44] Slovacek S. Whittinghill J. Flenoury L. Wiseman D. (2012). Promoting minority success in the sciences: the minority opportunities in research programs at CSULA. *J. Res. Sci. Teach.* 49 199–217. 10.1002/tea.20451

[B45] Sowell R. Allum J. Okahana H. (2015). *Doctoral Initiative on Minority Attrition and Completion.* Washington: Council of Graduate Schools.

[B46] Stanford F. C. (2020). The importance of diversity and inclusion in the healthcare workforce. *J. Natl. Med. Assoc.* 112 247–249. 10.1016/j.jnma.2020.03.014 32336480 PMC7387183

[B47] Taylor Z. W. (2019). Inarticulate transfer: do community college students understand articulation agreements? *Commun. Coll. J. Res. Pract.* 43 65–69. 10.1080/10668926.2017.1382400

[B48] Trapani J. Hale K. (2019). *Higher Education in Science and Engineering. Science & Engineering Indicators 2020.* Alexandria: National Science Foundation.

[B49] U.S. Census Bureau [USCB] (2012). *Profile America: Facts for Features.* Available Online at: https://www.census.gov/newsroom/releases/archives/facts_for_features_special_editions/cb12-ff19.html

[B50] U.S. Department of Education [USDE] (2012). *Hispanics and STEM Education.* Washington: U.S. Department of Education.

[B51] Valencia R. R. (2010). *Dismantling Contemporary Deficit Thinking: Educational Thought and Practice.* New York: Routledge.

[B52] Wang X. (2012). *Modeling Student Choice of STEM Fields of Study: Testing a Conceptual Framework of Motivation, High School Learning, and Postsecondary Context of Support. WISCAPE Working Paper*. Retrieved from ERIC (ED 529700). Madison: Wisconsin Center for the Advancement of Postsecondary Education.

[B53] Winterer E. R. Froyd J. E. Borrego M. Martin J. P. Foster M. (2020). Factors influencing the academic success of Latinx students matriculating at 2-year and transferring to 4-year US institutions–implications for stem majors: a systematic review of the literature. *Int. J. STEM Educ.* 7:34. 10.1186/s40594-020-00215-6

[B54] Yosso T. J. (2005). Whose culture has capital? a critical race theory discussion of community cultural wealth. *Race Ethn. Educ.* 8 69–91.

